# Photothermal-gas combination therapy promotes checkpoint blockade immunotherapy in colon cancer

**DOI:** 10.1080/14686996.2025.2504867

**Published:** 2025-08-27

**Authors:** Benchao Zheng, Hongbo Wang, Shiyi Zhai, Jiangsheng Li, Kuangda Lu

**Affiliations:** a Institute of Medical Technology, Peking University Health Science Center, Beijing, P. R. China; b Institute of Advanced Clinical Medicine, Peking University Health Science Center, Beijing, P. R. China; c Key Laboratory of Carcinogenesis and Translational Research of Ministry of Education, Key Laboratory for Research and Evaluation of Radiopharmaceuticals of National Medical Products Administration, Department of Nuclear Medicine, Peking University Cancer Hospital, Beijing, China

**Keywords:** Photothermal therapy, checkpoint blockade immunotherapy, nitric oxide, combination therapy, cancer

## Abstract

Checkpoint blockade immunotherapy emerges as a potential cure of cancer, but the monotherapy suffers from a low response rate in clinic. Photothermal therapy (PTT) that harvests light energy to ablate tumor is reported to activate tumor-specific immune response, meanwhile nitric oxide (NO) is considered to involve in immune regulation. Herein, we designed a multifunctional nanoplatform that enables photothermal-gas combination therapy by conjugating indocyanine green-thiol (ICG-SH) and s-nitrosoglutathione (GSNO) onto polyvinyl pyrrolidone (PVP)-coated gold nanoparticles (AIG). Upon near-infrared light (NIR) irradiation, AIG heats up the cancer cells and triggers NO release from GSNO, thus inducing apoptosis in the tumor. We found the combination of NO with photothermal treatment causes immunogenic cell death, which should synergize with checkpoint blockade immunotherapy. In the mouse colon cancer bilateral model, we observed complete eradication of light-irradiated tumors and suppression of distant untreated tumors in the AIG with anti-PD-1 (αPD-1) group. We detected significant increase of pro-inflammatory factors in serum, such as interferon- (IFN–γ), tumor necrosis factor-α (TNF-α) and interleukin-6 (IL-6) after PTT-gas-immunotherapy treatment, indicating the successful activation of the immune response. The improved immunogenicity caused by AIG with αPD-1 group allows for efficient antigen presentation, as evidenced by the increased infiltration of dendritic cells (DCs) into the tumor-draining lymph nodes (LNs). We also found promoted infiltration of CD8^+^ T cells in the untreated tumors in the AIG with αPD-1 group comparing to αPD-1 alone. Therefore, phototermal-gas-immune checkpoint blockade combination therapy represents a new promising treatment of metastatic cancer.

## IMPACT STATEMENT

We have constructed a novel nanoplatform (Au-ICG-GSNO) for photothermal-gas combination therapy of tumor, which synergize with checkpoint blockade immunotherapy to induce abscopal effect on murine colon cancer models.

## GRAPHICAL ABSTRACT

**Figure:**
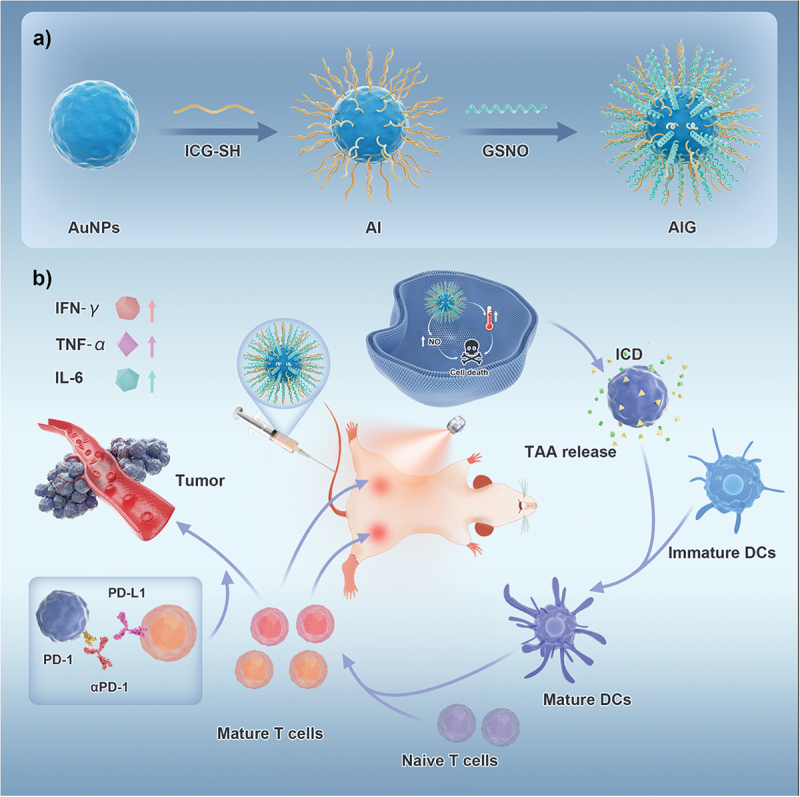

## 1. Introduction

Cancer has emerged as one of the leading threats to human health in modern society, contributing to high rates of morbidity and mortality globally [1–3]. Nowadays, the methods of treating tumor include chemotherapy, radiotherapy, surgery and so on [4–6]. However, treatment results often do not meet expectations due to the following reasons: (1) metastasis or recurrence of the tumor, (2) limited radiotherapy effect, and (3) drug resistance and toxic side effects of chemotherapy [7–9]. How to improve the effectiveness of cancer treatment and solve the current problems is still a huge challenge.

As a new treatment method, gas therapy has gained wide attentions in recent years [10–12]. Several kinds of gaseous molecules, such as nitric oxide (NO), hydrogen sulfide (H_2_S), and carbon monoxide (CO) play a very important part as messengers in a variety of biological processes, which can induce some physiological changes in cell, tissue, or organism [13]. NO can directly kill cancer cells and accelerate the process of cell apoptosis through oxidative/nitrosation of mitochondria and DNA in high concentrations, thereby has high potential for cancer therapy [14–16]. For instance, Shi et al. engineered an acid-activated NPs based on a pH responsive charge reversal triblock copolymer for the production of NO to achieve favorable penetration capability and antitumor activity [17]. However, the accurate delivery of NO to tumor remains a challenge. The thermal instability of many NO donors can be exploited to design thermosensitive delivery systems. Fang et al. introduced a gas/phototheranostic nanocomposite that integrates NIR-II-peak absorbing agents with a thermal-sensitive NO donor for atraumatic osteosarcoma therapy [18]. Therefore, photothermal therapy (PTT), which converts light energy into heat for the thermal ablation of cancer cells and can act as a natural trigger for NO delivery.

Although PTT efficiently eliminates local primary lesions, its inability to control tumor metastasis and recurrence remains a concern [19–24]. Recent research claims that PTT can initiate antitumor immunity by triggering the release of tumor antigens and damage-associated molecular patterns (DAMPs) from necrotic tumor cells [24–27]. However, a relatively low immune response can be induced by PTT alone due to immune escape. Immune checkpoint blockade therapy, which utilizes a series of antibodies that can target the overexpressed T-cell suppressor checkpoint signaling pathways, is a promising method to solve the adaptive immune evasion in tumor [28–30]. Thus, it is considered to activate efficient systemic immune responses when combined with PTT, thus improving the antitumor response rates and increasing the efficacy in metastatic tumor.

**Scheme 1.**
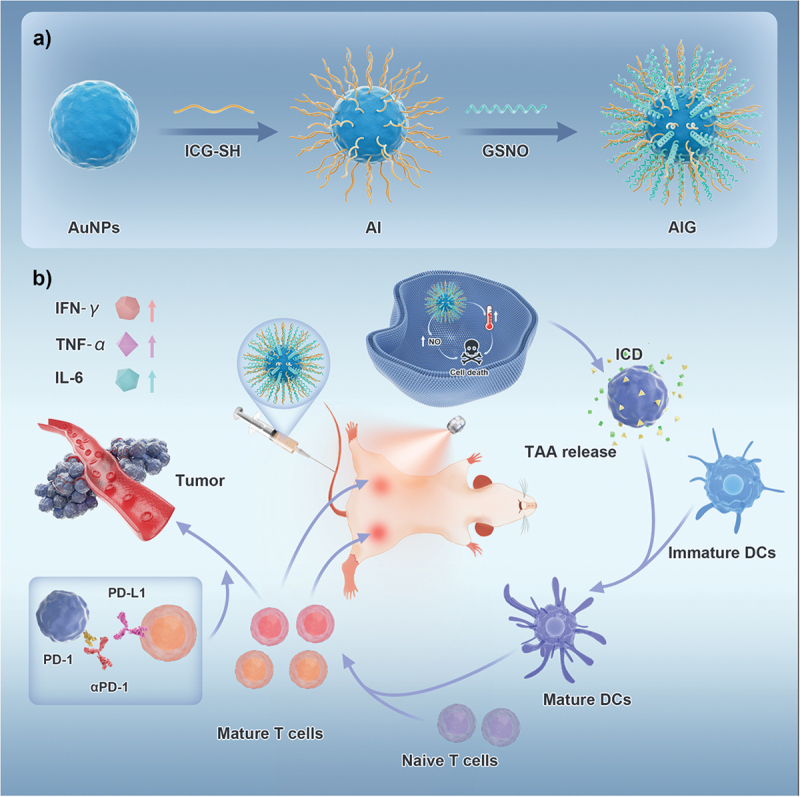
Schematic illustration of AIG nanoparticles for photothermal-gas combination therapy promotes checkpoint blockade immunotherapy. (a) The preparation of AIG NPs. (b) Mechanism of AIG NPs mediated PTT/NO combination therapy together with αPD-1 blockade for synergistically effective antitumor immunity.

In this study, we designed an Au-based multifunctional nanocarrier to realize near-infrared (NIR)-triggered PTT/NO cancer therapy. Polyvinylpyrrolidone (PVP)-coated gold nanoparticles (Au NPs) were conjugated with indocyanine green-thiol (ICG-SH) and S-nitrosoglutathione (GSNO), where ICG acts as an NIR photothermal agent and GSNO is a natural NO donor. The nano-carriers were designated Au-ICG-GSNO (AIG). Under NIR laser irradiation, AIG displayed a significant photothermal effect and exhibited effective controlled release of NO gas. The controlled release of a large amount of NO with PTT can further kill tumor cells and activate systemic immune effects. After PTT, with the help of programmed cell death protein 1 antibody (αPD-1), primary tumors can be effectively eliminated, and the released tumor associated antigens can stimulate a strong systemic antitumor immune response, which has an abscopal effect in inhibiting the growth of non-irradiated distant tumors in the MC38 bilateral model. In vivo experiments also indicated that PTT in combination with αPD-1 blockade therapy has significant efficacy in PTT-activated cancer immunotherapy (Scheme 1). In summary, the designed AIG nanocarriers provide a new approach for enhancing cancer therapeutic efficiency by achieving a synergistic effect between PTT/immunotherapy and NO gas.

## 2. Material and methods

### 2.1. Materials

HAuCl_4_·3 H_2_O (99%) and PVP K30 were purchased from Shanghai Bide Pharmaceutical Technology Co. Ltd. Indocyanine Green-Thiol (ICG-SH) were purchased from Xi ‘an Haoran Biotechnology Co. Ltd. S-nitrosoglutathione (98%) was purchased from Rhawn. Ascorbic acid (99.8%) was purchased from Beijing Tongguang Fine Chemical Co. All reagents were of analytical grade and used without further purification.

### 2.2. Characterization

Transmission electron microscopy (TEM) images were obtained using a JEM-1400 microscope. Dynamic light scattering (DLS) measurements were performed using a Zetasizer NanoZS instrument (Malvern, UK). UV-vis absorption spectra were obtained using a double-beam UV-vis spectrophotometer (T3202S, Youke Instrument, Shanghai). An 808 nm diode laser (Beijing Laserwave OptoElectronics Tech. Co., Ltd.) with tunable output power densities was used to study the photothermal effect. Fluorescent images of MC38 cells were obtained using a Zeiss LSM 900 confocal laser scanning microscope. Au concentration was assessed using a PerkinElmer NexlON 350X inductively coupled plasma optical emission (ICP-MS). Infrared thermal images were acquired by using a fluke infrared (IR) thermal camera. Flow cytometric analysis was performed using a CytoFLEX nano flow cytometer (Beckman, U.S.A.).

### 2.3. Preparation of AuNPs

First, Au crystalline nuclei were prepared [31]. NaBH_4_ (4 mg/mL, 0.6 mL) with 4 °C cold water was added to 3 mL of aqueous solution of 2.06 mg HAuCl_4_·3 H_2_O, and the mixture was stirred for 30 min. Next, 50 μL of the aforementioned solution was added to 6 mL of an ethylene glycol/H_2_O (5:1) solution with 100 mg of PVP, 20 mg of ascorbic acid, and 5 mg of HAuCl_4_·3 H_2_O. The resulting solution was stirred at room temperature for 2 h. The Au nanoparticles were then retrieved by ultrafiltration (Millipore, MWCO = 3 K) and washed three times with water.

### 2.4. Preparation of Au-ICG (AI)

The ICG-SH methanol solution was added to the AuNPs solution and the mixture was stirred for 2 h to obtain AI. AI nanoparticles were collected by centrifugation (15000 rpm, 30 min) and washed with methanol and ultrapure water.

### 2.5. Preparation of Au-GSNO (AG)

The GSNO aqueous solution was added to the AuNPs solution, and the mixture was stirred for 2 h to obtain AG. AG nanoparticles were collected by centrifugation (15000 rpm, 30 min) and washed twice with ultrapure water.

### 2.6. Preparation of Au-ICG-GSNO (AIG)

First, ICG-SH was added to the AuNPs solution and the mixture was stirred for 2 h to obtain AI. The AI nanoparticles were washed once with methanol and ultrapure water, and collected by centrifugation (15000 rpm, 30 min). Then, the AI was dispersed in an aqueous solution. Subsequently, GSNO was added to the aforementioned AI aqueous solution, and the mixture was stirred for 2 h. The solution was then washed twice with water and collected by centrifugation (15000 rpm, 30 min). The AIG was obtained.

### 2.7. Photothermal performance analysis of AIG

To investigate the photothermal performance of the as-synthesized AIG, an 808 nm NIR laser was delivered perpendicularly through 1.5 ml centrifuge tubes containing an aqueous dispersion (0.5 mL) of AIG at Au concentrations of 0, 12.5, 25, 50 and 100 µg/mL, which corresponds to 0, 6.5, 13, 26, 52 μM of ICG. The NIR laser light source had a power of 1 W/cm^2^ and illumination time of 10 min. The temperature was recorded every 20 s using an infrared thermal camera (225S-L24, Fotric, China).

For the photothermal stability tests, an aqueous solution of AIG (100 µg/mL) was exposed to 808 nm laser irradiation (1 W/cm^2^) for 10 min. The laser was then switched off for 10 min. The solution was irradiated for six on-off cycles. The temperature of the solution was recorded every 20 s.

Measurement of photothermal conversion efficiency was conducted according to a previous work [32].

### 2.8. Detection of NO release in the aqueous solution

DAF-FM DA (Beyotime, China) was mixed with the samples in aqueous solutions (Au: 10 μg/mL, DAF-FM DA: 5 μM), which were subsequently irradiated with an 808 nm laser (1 W/cm^2^) for different times (0, 2.5, 5, 7.5 and 10 min). After laser irradiation, the fluorescence spectra were recorded using a fluorescence spectrophotometer with an excitation wavelength of 485 nm and an emission wavelength of 515 nm. The fluorescence intensity at 515 nm was recorded at various time points.

### 2.9. Cell culture

MC38 cells were purchased from Wuhan Pricella Biotechnology Co. Ltd. (Wuhan, China). The above cells were cultured in Dulbecco’s modified Eagle’s medium, High Glucose (culture medium containing 10% fetal bovine serum (FBS) and 1% penicillin/streptomycin) at 37 °C with 5% CO_2_ in a Forma 3111 CO_2_ incubator (Massachusetts, U.S.A.).

### 2.10. Cellular uptake

The cellular uptake of AG, AI and AIG was evaluated in MC38 cells. Using 6-well plates, cells were seeded at 1.0 × 10^6^/well and cultured for 24 h. They were then added to the cells at a concentration of 10 μg/ml. After incubation for 1, 3, 6, 12 and 24 h, the cells were rinsed three times with PBS to remove excess nanoparticles, trypsinized and collected. Cell numbers were counted using a hemocytometer. Cells were digested with Lefort aqua regia and the Au concentrations were determined by ICP-MS (PerkinElmer, U.S.A.). The results are expressed as the amount of Au (ng) per 10^5^ cells.

### 2.11. Detection of intracellular NO release

MC38 cells were seeded in 35 mm laser confocal Petri dishes and incubated with high glucose complete DMEM (containing 10% FBS) for 24 h. The cells were incubated with AG, AI and AIG at a concentration of 100 μg/mL for 6 h and then washed three times with PBS to remove free drugs. The cells were then irradiated with an 808 nm laser (1 W/cm^2^) for 10 min, and cells not exposed to the laser served as a negative control. After that, the cells were incubated with DAF-FM DA solution (5 μM) at 37 °C for 20 min. Cells were washed twice with PBS and 1 mL of fresh DMEM was added to the cells. Finally, the cells were imaged using a confocal laser scanning microscope (CLSM).

### 2.12. Cell viability assay

MC38 cells were seeded in 96-well plates at 5000 cells per well and cultured in DMEM medium with 10% FBS at 37 °C with 5% CO_2_ for 24 h. Different concentrations (0, 100, 200, 300 and 400 μg/mL Au, corresponding to 0, 52, 104, 156, 208 μM ICG) of AG, AI and AIG dispersed in the culture media were added and co-incubated with the cells for 6 h. The cells were rinsed three times with PBS to remove the remnant nanoparticles. They were then irradiated with an 808 nm laser (1.0 W/cm^2^, 10 min). After 4 h, we incubated the cells with fresh medium containing 10% cck-8 solutions (Beyotime, China) for another 1 h. The absorbance of the CCK-8 solution in the cells was measured using a Microplate Reader (DeTie Biotechnology, China) at 450 nm.

### 2.13. Cytochrome c release

MC38 cells were seeded in 35 mm laser confocal Petri dishes for 24 h and then treated with PBS, AG, AI or AIG at an equivalent dose of 300 μg/mL for 6 h. The cells were then rinsed with PBS three times to remove free nanoparticles, followed by irradiation with an 808 nm laser (1.0 W/cm^2^, 10 min). After 4 h, cells were then stained with MitoTracker Red (Beyotime, China) at a concentration of 100 nM at 37 °C for 30 min. And fixed with 4% paraformaldehyde (PFA), blocked with 10% BSA, and then incubated with anti-cytochrome c antibody (ab133504, Abcam) overnight at 4 °C, followed by staining with Alexa Fluor®488-conjugated anti-rabbit secondary antibody (ab150073, Abcam) at room temperature for 2 h and Hoechst 33258 (Beyotime, China) respectively. Subsequently, the release of cytochrome c was observed by CLSM.

### 2.14. Ecto-calreticulin (CRT) staining on cell surface

MC38 cells were cultured in 35 mm laser confocal Petri dishes for 24 h and then treated with PBS, AG, AI or AIG at an equivalent dose of 300 μg/mL for 6 h. Cells were then rinsed with PBS three times to remove free nanoparticles and then exposed to 808 nm laser irradiation (1.0 W/cm^2^, 10 min). After another 4 h incubated, the cells were fixed in 4% paraformaldehyde, blocked with 10% BSA, and then incubated with anti-calreticulin (ab2907, Abcam) antibody overnight at 4 °C. Subsequently, cells were stained with Alexa Fluor®488-conjugated anti-rabbit secondary antibody (ab150073, Abcam) at room temperature for 1 h. After washing three times with PBS, the nuclei were stained with Hoechst 33258 for 20 min at room temperature and imaged using CLSM.

### 2.15. High mobility group box-1 (HMGB1) protein staining in the nuclear of cell

MC38 cells were cultured in 35 mm laser confocal Petri dishes for 24 h and then treated with PBS, AG, AI or AIG at an equivalent dose of 300 μg/mL for 6 h. Cells were rinsed with PBS three times to remove free nanoparticles and then exposed to 808 nm laser irradiation (1.0 W/cm^2^, 10 min). After another 4 h incubated, the cells were fixed in 4% paraformaldehyde, blocked with 10% BSA, and then incubated with anti-HMGB1 (ab18256, Abcam) antibody overnight at 4 °C. Subsequently, cells were stained with Alexa Fluor®488-conjugated anti-rabbit secondary antibody (ab150073, Abcam) at room temperature for 1 h. After washing three times with PBS, the nuclei were stained with Hoechst 33258 for 20 min at room temperature, and HMGB1 levels were measured using CLSM.

### 2.16. Apoptosis assay by flow cytometry

MC38 cells were inoculated into six-well plates and incubated with high glucose complete DMEM (containing 10% FBS) for 24 h. Then, PBS, AG, AI and AIG at an equivalent Au dose of 300 μg/mL were added to fresh DMEM. After co-incubation with the mixed medium for 6 h, the medium was removed and the cells were rinsed with PBS three times to remove free nanoparticles. The cells were irradiated with an 808 nm laser (1 W/cm^2^) for 10 min or not, and the cells were further cultured for 4 h. Subsequently, they were detached using trypsin and collected in 1.5 ml tubes. The Annexin V-FITC/PI apoptosis kit was used according to the manufacturer’s instructions for subsequent experiments. Finally, the cells were resuspended in PBS and immediately analyzed using a flow cytometer (Beckman, U.S.A.). Cells without any stain were used as negative controls.

### 2.17. Western blotting

MC38 cells (2 × 10^5^) were cultured in six-well plates for 24 h and then treated with PBS, AG, AI, or AIG at an equivalent Au dose of 100 μg/mL for 6 h. Cells were washed three times with PBS, followed by irradiation with an 808 nm laser (1 W/cm^2^) for 10 min. After 4 h, cells were lysed and used for the standard analysis of western blotting. After membrane transfer, anti-cleaved caspase-3 (ab214430, Abcam), anti-Bcl-2 (ab182858, Abcam), β-actin (66009–1-Ig, Proteintech), goat anti-mouse IgG secondary antibody HRP (ab205719, Abcam), and goat anti-rabbit IgG H&L (HRP) (ab97051, Abcam) were incubated separately with the cropped membranes for blotting.

### 2.18. Animal model

C57BL/6N mice (5–6 weeks old, female) were purchased from Beijing Vital River Laboratory Animal Technology Co., Ltd. (China). All animal experiments were performed with permission from the Animal Ethics Committee of Beijing Vital River Laboratory Animal Technology Co., Ltd. (China) according to the guidelines approved by the Beijing Administration of Experimental Animals. The cell line MC38 (2 × 10^6^ cells) in 100 μL PBS was injected subcutaneously into the right flank of C57BL/6N mice. When the tumor volume reached approximately 50–100 mm^3^, the mice were ready for experiments.

### 2.19. Photoacoustic imaging (PAI) performance of AIG

For in vivo PA imaging, MC38 tumor – bearing C57BL/6N mice were anesthetized and monitored using a photoacoustic/ultrasonic multimodal small-animal imaging system (Vevo 3100 LAZR, Canada). PA imaging was performed after the injection of AIG (10 mg Au/kg body weight) at different time points (1, 6, 12, 24 and 48 h), and PBS was injected as the control. The entire mouse was scanned at a step size of 0.3 mm. After data acquisition, PA images were reconstructed using a standard back-projection algorithm.

### 2.20. Evaluation of blood half-lives and biodistribution study of AIG

MC38 tumor-bearing mice (*n* = 3) were intravenously injected with 200 μL AIG (10 mg Au/kg body weight). Blood was extracted from the angular vein at different time points (5 min, 30 min, 1, 3, 6, 12, 24 and 48 hours). At each time point, the mice were euthanized, and the main organs (heart, liver, spleen, lungs and kidneys) and tumors were excised and weighed. All the samples were decomposed using Lefort aqua regia. The Au content of the samples was determined using ICP-MS. The results are expressed as %ID/g, which was calculated as the percentage of the element mass per gram of tissue to the total injected element mass.

Using Origin software, the blood half-life of AIG (%ID/g) was fitted using a two-compartment intravenous injection model.

### 2.21. In vivo antitumor efficacy

All animal experiments were approved by the Laboratory Animal Center of Peking University. The mice were randomly divided into eight groups (*n* = 6 for each group) and treated with the following experimental conditions: (1) PBS (2) AG (3) AI (4) AIG (5) PBS +808 nm laser (6) AG +808 nm laser (7) AI +808 nm laser (8) AIG +808 nm laser. Different groups, at an equivalent dose of 10 mg/kg body weight or PBS, were injected intravenously into the tumor-bearing mice. Twelve hours after injection, the mice were anesthetized and the tumors were exposed to an 808 nm NIR laser (1 W/cm^2^) for 10 min. After these treatments, tumor size was measured using a caliper every other day and calculated as volume = (tumor length) × (tumor width)^2^/2. The body weights of the mice were also recorded and the results are shown as a function of time. At the end of the experiment, the major organs (heart, liver, spleen, lung and kidneys) were excised and embedded in paraffin for hematoxylin and eosin (H&E) staining. And tumors were photographed, weighted and collected for H&E, Tunel, Ki-67, CRT and HMGB1 immunofluorescence staining.

### 2.22. Abscopal effect on bilateral tumor model

MC38 bilateral tumor models were established in C57BL/6N mice by subcutaneously inoculating 2 × 10^6^ MC38 cells into the right flank (primary tumor) and 4 × 10^5^ MC38 cells into the left flank (distant tumor). When the primary tumors reached approximately 100 mm^3^, mice were randomly divided into four groups (*n* = 6): (1) PBS without irradiation as a control; (2) AIG plus αPD-1 without irradiation; (3) AIG with irradiation; (4) AIG with irradiation plus αPD-1. They were injected into the mice via tail vein at the doses of 10 mg/kg b.w. Twelve hours after injection, the primary tumors were irradiated with an 808 nm laser at 1.0 W/cm^2^, and αPD-1 was intraperitoneally injected at a dose of 100 μg/mouse every two days until the total dose is 200 μg per mouse. Tumor volume and body weight was measured every two days following treatment. When the primary tumor size of the PBS group reached 1000 mm^3^, mice were sacrificed and both the primary and distant tumors were excised, photographed and weighed. And the blood was collected for the detection of related cytokines (Mouse interleukin 6 (IL-6), tumor necrosis factor α (TNF-α), and interferon γ (IFN-γ)).

### 2.23. Flow cytometry for immune response

Treated MC38 tumor-bearing C57BL/6N mice were sacrificed, distant tumors were collected, cut into pieces, treated with 2 mg/mL collagenase I and IV (Macklin, China) for 30 min at 37 °C, and ground with the rubber end of a syringe through 70 μm nylon mesh filters. Bilateral tumor-draining lymph nodes (LNs) were harvested and ground using 70 μm nylon mesh filters to obtain a single-cell suspension. Cells were then stained with the following fluorochrome-conjugated antibodies: CD45.2 (104.2), CD3ε (145-2C11), CD8α (53–6.7), CD11b (M1/70), CD11c (N418), CD86 (GL-1), CD80 (16-10A1) and subjected to flow cytometry.

### 2.24. Statistical analysis

All results are shown as the mean ± SD. Statistical analysis was conducted using the Student’s two-sided t-test. *p < 0.05, **p < 0.01, ***p < 0.001. Origin 2023 software was used for statistical analyses.

## 3. Results and discussion

### 3.1. Preparation and characterizations of AIG

**Figure 1.**
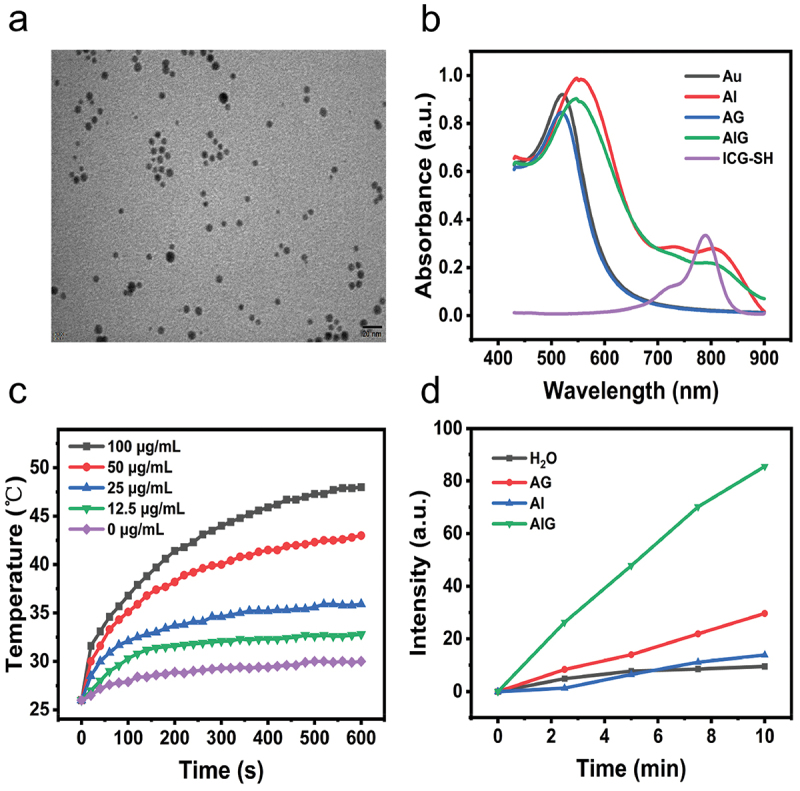
(a) Transmission electron microscopy (TEM) pictures of AIG. Scale bar: 20 nm. (b) UV/Vis absorption spectra of Au, AG (Au-GSNO), AI (Au-ICG), AIG (Au-ICG-GSNO) and ICG-SH. (c) Temperature variation curves of AIG solutions with different concentrations (0, 12.5, 25, 50, 100 μg/mL by Au or 0, 6.5, 13, 26, 52 μM by ICG) under 808 nm laser (1.0 W/cm^2^). (d) *In vitro* NO release from different nanoparticles in a PBS buffer (pH 7.4) as a function of time under 808 nm laser irradiation (1.0 W/cm^2^).

The synthesis of the Au NPs (Au) is described in the Experimental Section. Subsequently, ICG-SH and GSNO were conjugated to Au NPs in two steps through stable Au-S bonds, and AIG nanoparticles were synthesized. For subsequent experiments, we synthesized Au-GSNO (AG) and Au-ICG (AI) nanoparticles as control groups. Transmission electron microscopy (TEM) indicated that the AIG particles were spherical with a diameter of approximately 10 nm (Figure 1a). After 24 hours of dispersion in PBS, the size and morphology of AIG kept unchanged by TEM image observation (Figure S1), indicating that AIG was stable in the physiological environment. The hydrodynamic diameter of Au NPs was about 22 nm with a PDI of 0.31 (Figure S2). After ICG and GSNO loading, the hydrodynamic diameter of the nanoparticles increased to approximately 64 nm, with a PDI of 0.20. Moreover, the surface charge of the nanoparticles also changed from −4.95 ± 0.54 mV to −19.73 ± 0.67 mV (Figure S3). UV-visible absorption spectroscopy revealed that both AI and AIG exhibited a significant absorption peak at 805 nm, which correlated with the absorption peak (~795 nm) of the ICG molecules (Figure 1b). Therefore, it was demonstrated that the ICG molecules were coupled to the surface of the gold nanoparticle successfully. Meanwhile, compared with the free ICG, AI and AIG nanoparticles showed a red-shift in the absorption spectra, which can be attributed to the electron delocalization promoted by noncovalent interactions [33,34]. The payloads of ICG and GSNO were evaluated by UV-vis spectroscopy (absorption peaks at 785 and 340 nm, respectively) [35,36]. According to the two standard curves (Figure S4 and S5), we measured the free ICG and GSNO molecules in the supernatant after centrifugation, the loading percentages of ICG and GSNO were individually 33.83% and 10.33% by subtraction method. The Au content of the AIG samples was determined using inductively coupled plasma mass spectrometry (ICP-MS). The molar ratio of Au:ICG:GSNO was 160:18:5, respectively (Table S1).

Photothermal conversion efficiency is vital for PTAs. There was an obvious increase in temperature when the AIG was exposed to 808 nm laser irradiation (1 W/cm^2^). With increasing concentration, the final temperature and heating rate increased. The maximum temperature arrived at 48 °C after 10 minutes irradiation (100 μg/mL by Au or 52 μM by ICG concentration; Figure 1c). In addition, the AIG exhibited high photostability. When the AIG was subjected to NIR laser irradiation for six cycles, its photothermal performance remained almost unchanged (Figure S6). Photothermal conversion efficiency (η) is an essential parameter used to evaluate photothermal agents. The η value of AIG was calculated to be 38.3% with 808 nm laser irradiation (Figure S7). Next, we investigated NIR-triggered NO release from the AIG. The NO content of the solution upon NIR laser irradiation was quantified using a commercial fluorescent probe, DAF-FM DA. The NO release profiles of different samples under laser irradiation are shown in Figure 1d. For the AI and water groups without GSNO loading, there was only a very weak increase in the fluorescence intensity. In contrast, NO was quickly released from the AIG under NIR laser irradiation, and the amount of NO released increased with increasing irradiation time, indicating that controllable release of NO could be realized by NIR laser irradiation.

### 3.2. Cellular NO production and cytotoxicity studies

**Figure 2.**
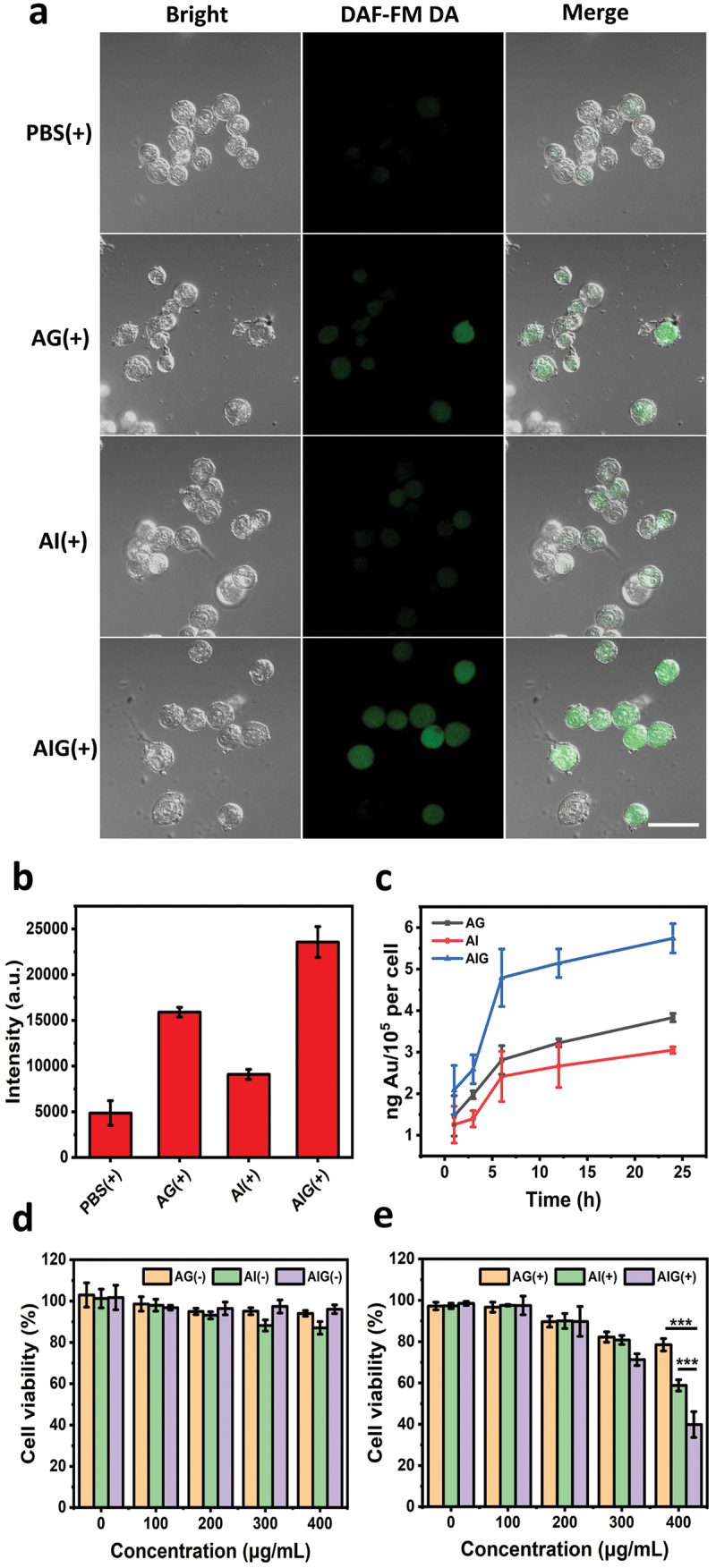
(a) CLSM images of intracellular NO release in MC38 cells incubated different nanoparticles under NIR laser irradiation. Scale bar: 40 μm. (b) Average cellular fluorescence signal of DAF-FM DA indicating NO production under 808 nm laser irradiation in each group. (c) Cellular uptake amounts of AG, AI and AIG in MC38 cells at different incubation time. (d) Cell viabilities of MC38 cells incubated with AG, AI and AIG at different concentrations (0, 100, 200, 300 and 400 μg/mL) in dark. (e) Cell viabilities of MC38 cells incubated with AG, AI and AIG at different concentrations (0, 100, 200, 300 and 400 μg/mL) under 808 nm laser irradiation. *p < 0.05, **p < 0.01, and ***p < 0.001.

The NO production ability of different NPs in MC38 cells was examined using the intracellular NO probe DAF-FM DA and observed using a fluorescence microscope. Both the fluorescence images and quantitative data demonstrated that the AIG + laser group exhibited a significantly higher level (*p* < 0.01) of green fluorescence than those in the AI + laser and PBS + laser groups (Figure 2a, and b). The fluorescence intensity in the AG + laser group was significantly lower than that in the AIG group (*p* < 0.05). No obvious fluorescence was observed in the dark controls (Figure S8). NIR laser irradiation can slightly increase NO release from GSNO, however, high temperatures still play a more dominant role.

The uptake of AIG by MC38 cells was studied to determine the appropriate incubation time for subsequent cell experiments throughout this research. As shown in Figure 2c, with an increase in incubation time, the amount of Au in the cells was almost full at approximately 6 h. Therefore, we incubated cells with samples for 6 h in the following experiments unless otherwise stated.

For the *in vitro* cytotoxicity study, dose-related cytotoxicity was determined using a CCK-8 assay. The cytotoxicity of different NPs at different concentrations (0, 100, 200, 300, 400 μg/mL by Au or 0, 52, 104, 156, 208 μM by ICG) in the dark or irradiated for 10 min (808 nm laser, 1 W/cm^2^) was investigated. None of the groups in the dark exhibited any obvious cytotoxicity even at high concentrations, which proved the biocompatibility of the nanocarriers (Figure 2d). After NIR laser irradiation, the viability of MC38 cells began to decline with increasing incubation concentration. AI treatment with NIR irradiation had a significant killing effect on cells (*p* < 0.001) compared with the AG group, especially at a high concentration of 400 μg/mL, which proved the photothermal effect of AI. The cell viability of the AIG group was significantly lower than that of the AI group (*p* < 0.001), indicating that the combination of NO and PTT was more effective than PTT alone in decreasing the viability of cancer cells (Figure 2e).

### 3.3. Cell death mechanism and immunogenic cell death (ICD) analysis

**Figure 3.**
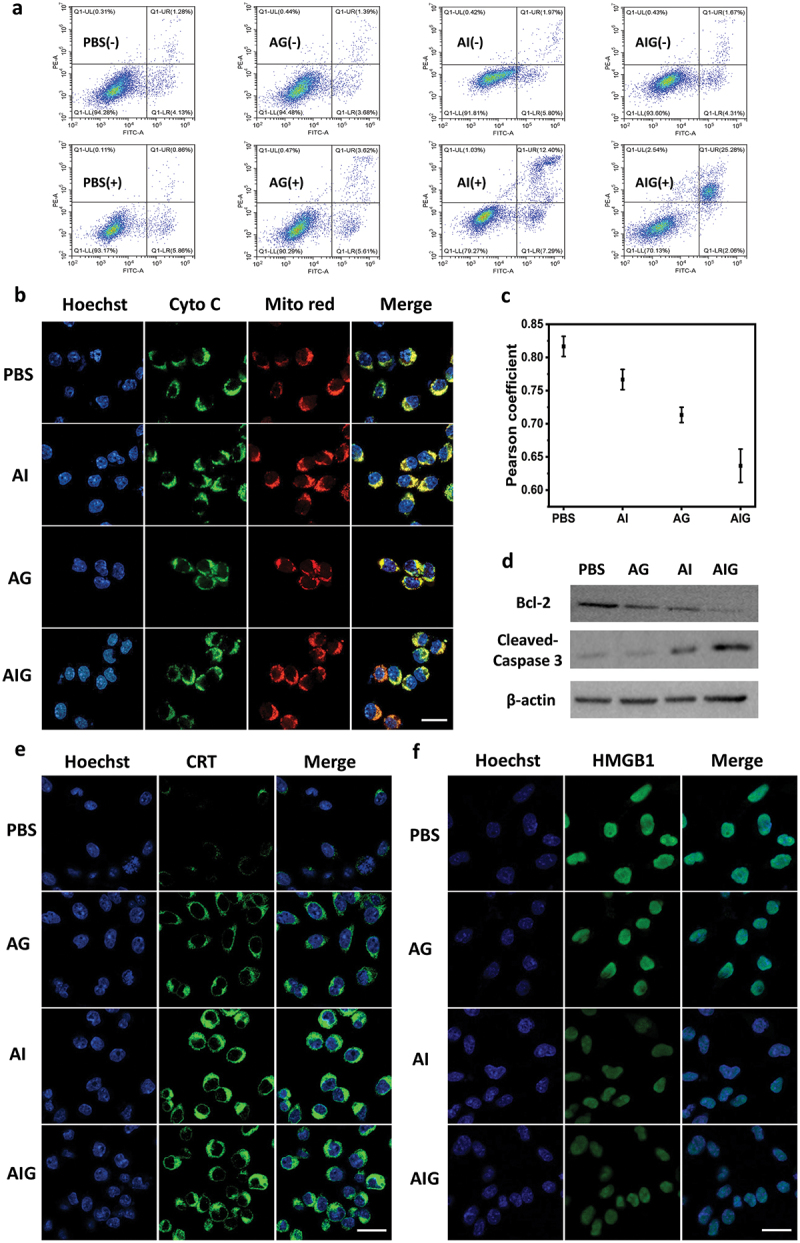
(a) Flow cytometry analysis showing cells apoptosis after different treatments. (+) refers to 808 nm laser irradiation. (b) CLSM images showing cyto C release. Scale bar: 40 μm. (c) Pearson correlation analysis indicating the co-localization of mitochondria and cyto c in different samples. (d) Western blot analysis of Bcl-2 and cleaved-caspase 3 in MC38 cells after irradiation with different groups. (e) Immunofluorescence staining showing CRT exposure on MC38 cells after different treatments. Scale bar: 40 µm. (f) Immunofluorescence staining of HMGB1 in the nucleus after different treatments. Scale bar: 40 µm.

It has been reported that NO could exert physiological effects, such as oxidative and nitrosative stress activation, causing mitochondrial dysfunction and DNA functional damage [37]. These effects lead to cell apoptosis, thereby realizing a potential synergistic therapeutic effect with PTT. We investigated the pattern of cell death in MC38 cells by flow cytometry. MC38 cells were treated with PBS, AG, AI or AIG with or without light irradiation. The cells were then stained with FITC-Annexin V/PI and detected using a flow cytometer. No obvious apoptosis was observed in any of the treatment groups in the absence of NIR laser irradiation. After NIR laser irradiation, the ratio of apoptotic cells (both early and late stage) treated AI increased to 19.69% compared to the AG and PBS negative control groups, which was mainly induced by the photothermal effect. The proportion of apoptotic cells treated with AIG further increased to 27.34%, especially the proportion (25.28%) of late apoptotic cells (Figure 3a). This was mainly attributed to the rapid release of NO from the AIG, which caused toxic effects on tumor cells. These results suggest that NO synergizes with PTT to destroy tumor cells more effectively.

The release of cytochrome c (Cyto c) is generally considered an indication of apoptotic cell death [38]. As shown in Figure 3b and S9, the AIG group had the least overlap of Cyto c and mitochondrial fluorescence compared with the other groups, indicating that more Cyto c was released into the cytoplasm in the AIG group. The Pearson coefficients for PBS, AI, AG, and AIG were 0.83, 0.77, 0.72 and 0.61, respectively (Figure 3c). These results also showed a significant disruption of mitochondria by AIG under NIR irradiation.

We further adopted western blotting (WB) to analyze proteins associated with apoptosis. Bcl-2 and cleaved caspase-3 levels after NIR laser irradiation were examined to probe the activation of mitochondria-associated apoptosis [39,40]. As illustrated in Figure 3d and S10, AIG-treated cells showed the greatest downregulation of Bcl-2 proteins, indicating greater mitochondrial outer membrane permeabilization in the AIG groups. In addition, we detected the highest level of cleaved-caspase-3 in the AIG treated group. These results suggest activation of the mitochondrial apoptotic pathway.

Calreticulin (CRT) in the endoplasmic reticulum is exposed on the surface of injured cancer cells, which is an important sign of ICD [41,42]. CRT expression on the MC38 cell surface was visualized by immunofluorescence staining after different treatments. Cells treated with both AI and AIG showed obvious green fluorescence, indicating CRT (Figure 3e). And the fluorescence intensity of AIG and AI groups was 2.4 and 2.56 times higher than that of PBS group, respectively (Figure S11a), indicating that PTT could significantly enhance CRT exposure due to enhanced cell apoptosis. As another ICD biomarker, high mobility group box 1 (HMGB1) in the nuclei is released into the extracellular space [43,44]. As shown in Figure 3f, attenuated green fluorescence in the nuclei indicates the release of HMGB1 from damaged cancer cells. The AIG with NIR laser irradiation groups achieved a 1.90-fold reduction in nuclear HMGB1 compared to the PBS groups based on the mean fluorescence intensity (Figure S11b). These results indicate that the AIG induces a strong ICD effect under NIR laser irradiation.

### 3.4. In vivo pharmacokinetics and biodistribution of AIG

To assess the pharmacokinetics and biodistribution of AIG, MC38 subcutaneous tumor xenografts were established. An equivalent dose of 10 mg/kg AIG was injected intravenously into the mice. The blood half-life and the biodistribution of AIG were analyzed using ICP-MS. Figure S12a shows that the elimination half-life (t_1/2β_) of AIG was approximately 4.5 h, indicating that AIG can accumulate at tumor sites. Biodistribution results showed that AIG could be also enriched in tumor sites and reached its maximum at 12 h, with a value of 3.5%ID/g. (Figure S12b).

**Figure 4.**
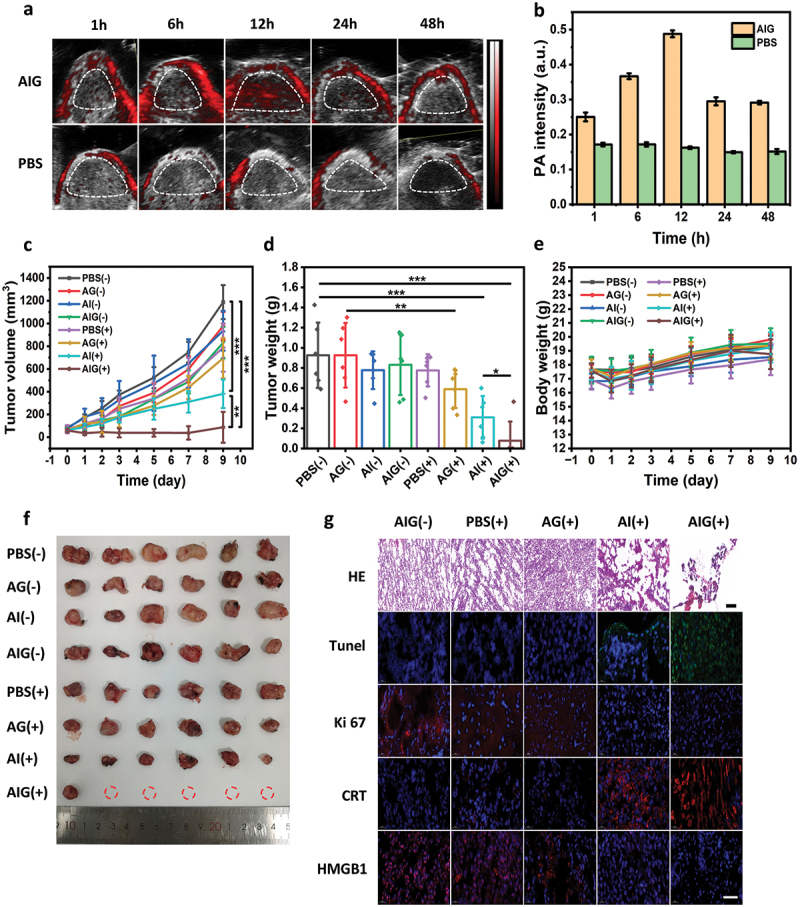
(a) PA imaging showing AIG accumulation over time in mice tumor. The dashed area corresponds to the tumor site. (b) Relative PA intensities in the tumor region. (c) Tumor growth curves of MC38 tumor-bearing mice treated with PBS, AG, AI or AIG by intravenous injection with (+) or without (-) NIR laser irradiation. (d) Average weights of excised tumors at the end point. (e) Body weights of mice after different treatments. (f) Photographs of the dissected tumors after different treatments. (g) H&E histological staining, scale bar: 100 μm; tunel immunofluorescence staining, Ki-67, CRT immunohistochemical staining and HMGB1 immunofluorescence staining of excised tumor slices for AIG (-), PBS (+), AG (+), AI (+) or AIG (+) treatment groups. Scale bar: 40 μm. *p < 0.05, **p < 0.01, and ***p < 0.001.

The photothermal performance and high photostability of AIG make it an ideal contrast agent for PAI applications. Therefore, we confirmed tumor accumulation of AIG with PAI. AIG was injected intravenously into tumor-bearing mice at a dose of 10 mg/kg and PBS was used as a negative control. As shown in Figure 4a and b, the acoustic intensities of the tumor sites increased with the extension of the injection time and reached a maximum at 12 h.

### 3.5. In vivo antitumor efficacy

MC38 subcutaneous tumor xenografts were established to investigate the anti-tumor activity of AIG *in vivo*. When the tumors reached approximately 100 mm^3^ in volume, PBS, AG, AI and AIG were injected intravenously at a dose of 10 mg Au/kg body weight per mouse. Twelve hours after administration, the tumors were exposed to an 808 nm NIR laser (1 W/cm^2^) for 10 min. As shown in Figure 4c,d and f AIG injection with NIR irradiation nearly cured the tumors at the end of treatment. The tumors were eradicated in five of the six mice. The average tumor sizes in the AIG with NIR laser treated group were only 7.2% of that in PBS group. In comparison, the tumor sizes in the AI with NIR irradiation group were 32.1% of those in PBS group, demonstrating less potent anticancer effect of photothermal damage alone. The weights of the stripped tumors in each group at the endpoint confirmed that the AIG with NIR irradiation group had the lowest tumor weight, which was significantly lower than those in the PBS (*p* < 0.001) and AI with NIR irradiation groups (*p* < 0.05). Based on the results of H&E staining, Tunel and Ki-67 immunofluorescence staining (Figure 4g), the AIG with NIR treatment group exhibited tumor tissues with disrupted morphology and structure, enhanced apoptotic signals, and significantly reduced proliferation rates compared to other groups. These findings suggest that AIG significantly induces apoptosis and inhibits tumor cell proliferation. The expression levels of representative ICD markers in tumor tissues, including cell surface exposure to CRT and HMGB1, were also determined. The AIG with NIR irradiation group had the greatest exposure to CRT and release of HMGB1, which again proved that the PTT/NO synergistic therapy could activate the immune response at the tumor site. During the treatment, both body weight and histological analysis with or without NIR laser irradiation in the major organs of mice showed negligible changes among all groups (Figures 4e, S13 and S14), proving the excellent biocompatibility of these nanoparticles. In addition, no obvious variation in behavior and organ functioning was detected in any group, suggesting that AIG nanoparticles with controlled photothermal release of NO via tail vein injection had no apparent systemic toxicity.

### 3.6. In vivo antitumor abscopal effect and immune response activation

**Figure 5.**
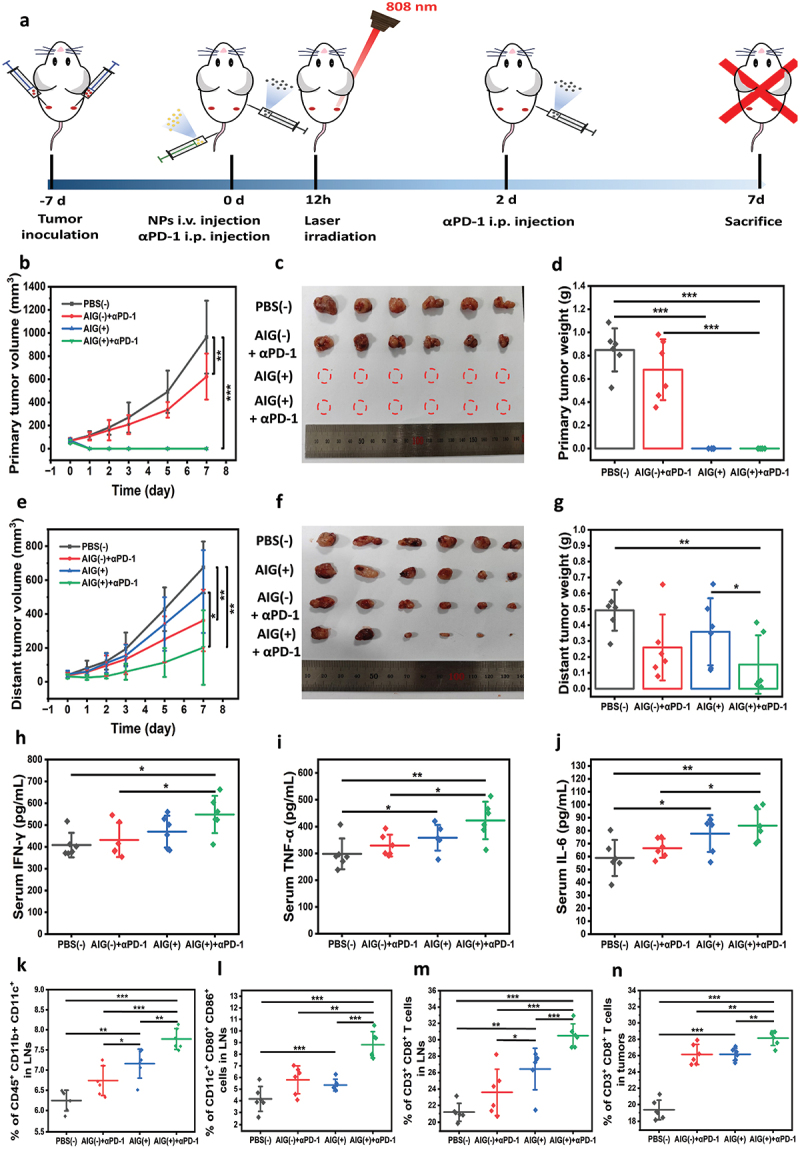
(a) Schematic illustration of animal experimental design. (b) Growth curves of the primary tumors in the MC38 bilateral models. Photographs (c) and weights (d) of dissected primary tumors at day 7 post-treatment. (e) Growth curves of distant tumors after different treatment. Photographs (f) and weights (g) of dissected distant tumors at day 7 post-treatment. The serum levels of IFN-γ (h), TNF-α (i) and IL-6 (j), after treatments (*n* = 6). The percentages of DCs (CD45^+^CD11b^+^CD11c^+^, k), maturated DCs (CD11c^+^CD80^+^CD86^+^, l), CD3^+^CD8^+^ T cells (m) in tumor-draining LNs after treatments (*n* = 6). (n) The percentages of CD3^+^CD8^+^ T cells in distant tumors after treatments (*n* = 6). *p < 0.05, **p < 0.01, and ***p < 0.001.

To determine whether the immune response induced in the primary tumor was strong enough to inhibit untreated distant tumors, a bilateral syngeneic MC38 tumor model was established. The mice were injected intravenously with different nanoparticles, and the primary tumors were either irradiated or not irradiated 12 h after injection, whereas distant tumors were shielded from irradiation (Figure 5a). As shown in Figure 5b–d, the primary tumors in the AIG with NIR laser and AIG with NIR laser plus anti-PD-1 (αPD-1) groups were completely eradicated after irradiation. PBS and αPD-1 alone exhibited no apparent inhibitory effect on primary tumors. For distant tumors, the combination of AIG with NIR irradiation and αPD-1 therapy induces a stronger abscopal effect than AIG with NIR irradiation alone. The average tumor sizes and weights in the AIG with NIR irradiation plus αPD-1 group were 201.10 ± 219.89 mm^3^ and 151.93 ± 184.2 mg, representing reductions of 70.3% and 69.2%, respectively, compared to the PBS group (Figure 5e–g). In contrast, the AIG with NIR group exhibited tumor sizes and weights of 532.60 ± 244.42 mm^3^ and 358.33 ± 210.70 mg, while αPD-1 alone group also demonstrated significant inhibition of distant tumors, with average tumor sizes and weights of 362.05 ± 182.18 mm^3^ and 259.49 ± 207.43 mg. Notably, four mice in the AIG with NIR plus αPD-1 group exhibited a remarkably stronger response, with an average tumor weight of 33.95 ± 18.07 mg, which was only 6.9% of that in the PBS group. This corresponded to a 66% synergistic response to the PTT/NO therapy combined with checkpoint blockade therapy. We propose that potent tumor destruction induced by PTT/NO treatment facilitates the release of tumor-associated antigens (TAAs), which promote T cell maturation and synergize with αPD-1 to enhance cellular immunity. Throughout the treatment process, the body weight of the mice in each group remained stable (Figure S15). Besides, H&E staining of the major organs further verified that negligible side effects of all samples were observed (Figure S16), indicating the good biosafety of AIG plus αPD-1.

Significantly higher serum levels of pro-inflammatory factors, including IFN-γ, TNF-α, and IL-6, were observed in mice treated with AIG with NIR laser plus αPD-1 compared with PBS groups (Figure 5h–j). Meanwhile, the levels of these pro-inflammatory factors also increased to some extent by AIG with NIR laser treatment, suggesting that AIG mediated PTT/NO combination therapy caused decent inflammatory response.

To explore the mechanism by which AIG enhances immunotherapy, we first studied the variations in antigen-presenting cells (APCs) in mice after treatment. Dendritic cells (DCs), as the most impactful professional APCs in vivo, have a potent capacity to process antigens and present them to T cells in the lymph nodes (LNs) to provoke antitumor immunity [45,46]. The enhanced infiltration of DCs may result from the improved immunogenicity caused by AIG with NIR laser plus αPD-1 and allow for efficient antigen capture and presentation (Figure 5k). Meanwhile, mature DCs expressed characteristic surface markers (CD80/CD86) on their cell surfaces, which were essential to activate later T-cell responses. Remarkably, the expression of CD11c^+^ CD80^+^ CD86^+^ was significantly enhanced in mice treated with AIG with NIR irradiation plus αPD-1 compared with PBS groups (*p* < 0.001), suggesting the further formation of mature DCs through the photothermally induced generation of tumor antigens with the assistance of αPD-1 (Figure 5l).

Antigen presentation can enhance T cell proliferation and activation, thereby strengthening the immune response to achieve tumor eradication [47–50]. To evident T-cell activation, the relative abundance of T cells in LNs and tumors was evaluated. The relative abundance of CD8^+^ T cells significantly increased in the tumor-draining LNs after AIG with NIR irradiation plus αPD-1 treatment (Figure 5m), indicating successful recruitment of T cells that facilitates antigen presentation. In addition, both AIG with NIR alone or αPD-1 alone had significantly higher CD8^+^ T cell infiltration at distant tumors (*p* < 0.001) compared with PBS group (Figure 5n), indicating that both damage-induced antigen presentation and checkpoint blockade contribute to tumor recognition. AIG with NIR irradiation plus αPD-1 further enhanced CD8^+^ T cell infiltration (*p* < 0.01, comparing to AIG with NIR and αPD-1 alone), thereby proving the synergy between PTT/NO treatment and checkpoint blockade therapy.

These results confirmed that AIG with laser irradiation enhanced DCs maturation and T cell infiltration through ICD effects, thereby inducing a robust anti-tumor immune response when combined with αPD-1 mediated immune checkpoint blockade.

## 4. Conclusion

In summary, a smart nanosystem (AIG) for PTT/NO combination therapy was constructed to achieve the controlled release of NO during photothermal therapy. This therapeutic approach induces immunogenic cell death (ICD) in tumor cells, thereby enhancing dendritic cells (DCs)-mediated antigen presentation and subsequent T cells activation. αPD-1 has been employed to mitigate the immunosuppressive effects of the tumor microenvironment and enhance T-cell infiltration. Therefore, AIG successfully eliminated primary tumors in MC38 tumor-bearing mice under NIR laser irradiation, further strengthening the suppression of distant tumors and activating systemic immunity in combination with αPD-1. This controllable strategy of photothermal/gas combination therapy can provide insights into the clinical treatment of tumor metastasis.

## Supplementary Material

Supplemental Material
