# Supplementary material for: Photothermal-gas combination therapy promotes checkpoint blockade immunotherapy in colon cancer
Source: Sci Technol Adv Mater. 2025 Aug 27;26(1):2504867. doi: 10.1080/14686996.2025.2504867 (PMC12392434; doi:10.1080/14686996.2025.2504867)
Supplement: Supplemental Material [file TSTA_A_2504867_SM6454.docx]

**Photothermal-Gas Combination Therapy promotes Checkpoint Blockade Immunotherapy in Colon Cancer**

Benchao Zheng^a, b^, Hongbo Wang^a, b^, Shiyi Zhai^a, b^, Jiangsheng Li^c^ and Kuangda Lu^a,^ b,*

^a^ Institute of Medical Technology, Peking University Health Science Center, Beijing

100191, P. R. China ^b^ Institute of Advanced Clinical Medicine, Peking University, Beijing 100191, P. R. China

^c^ Key Laboratory of Carcinogenesis and Translational Research of Ministry of Education, Key Laboratory for Research and Evaluation of Radiopharmaceuticals of National Medical Products Administration, Department of Nuclear Medicine, Peking University Cancer Hospital, Beijing 100142, China

*E-mail: lukuangda@hsc.pku.edu.cn


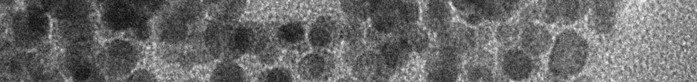

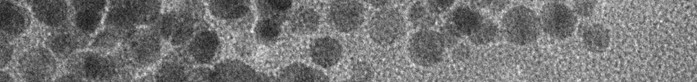

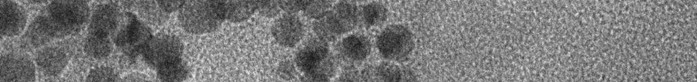

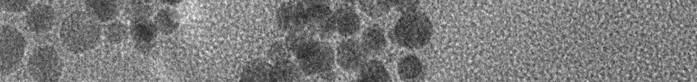

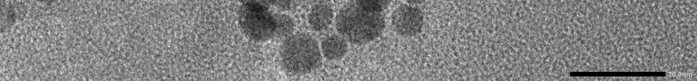


Figure S1. TEM pictures of AIG dispersed in PBS for 24 h. Scale bar: 20 nm.


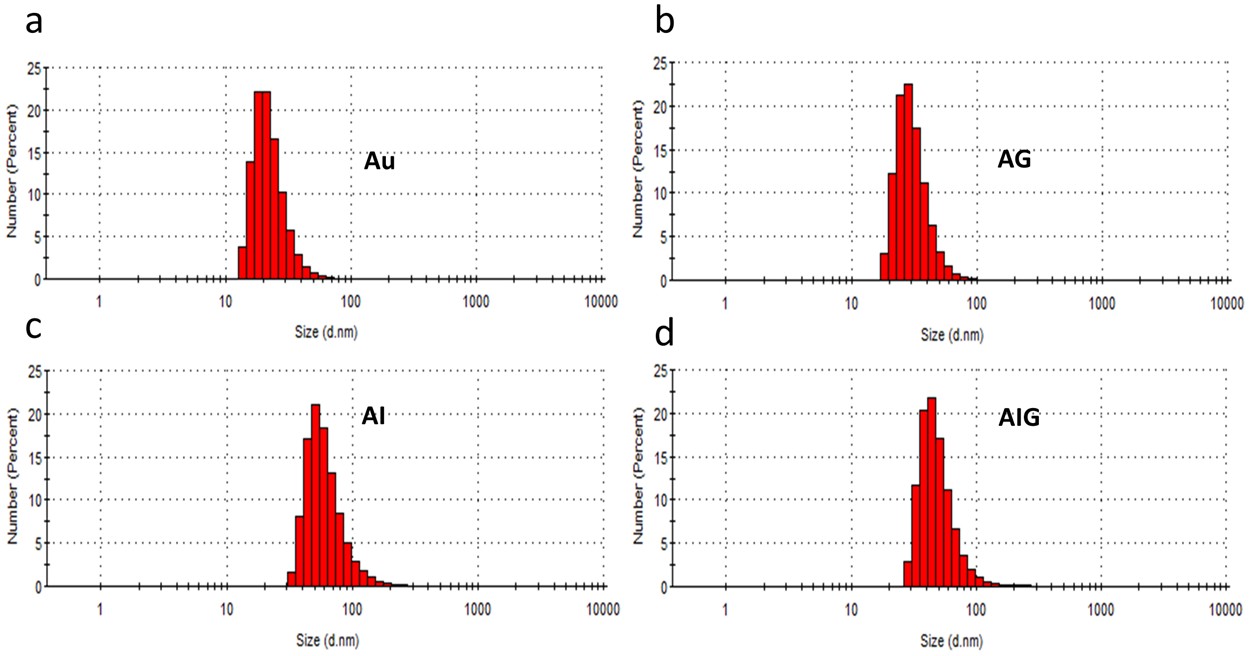


Figure S2. Hydrodynamic diameters of Au, Au-GSNO (AG), Au-ICG (AI) and Au-ICG-GSNO (AIG).


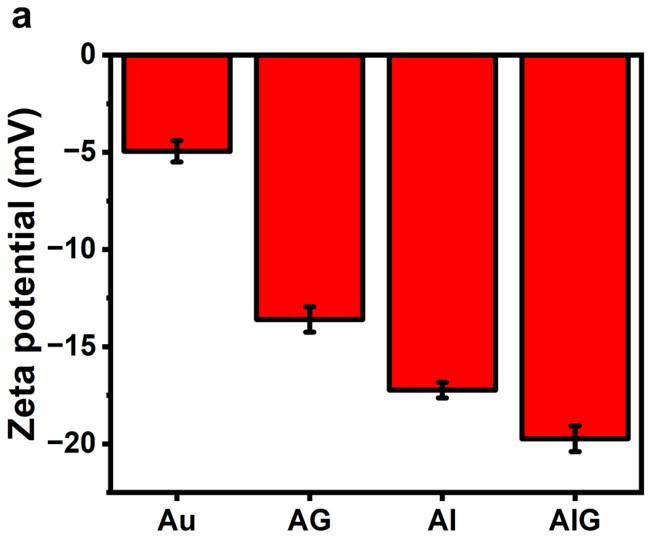


Figure S3. Zeta potential of Au, AG, AI and AIG.


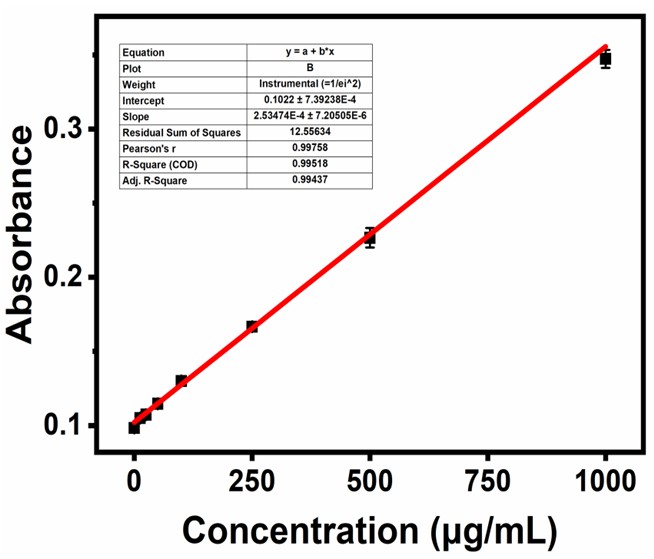


Figure S4. (a) The standard curve of GSNO according to the absorption.


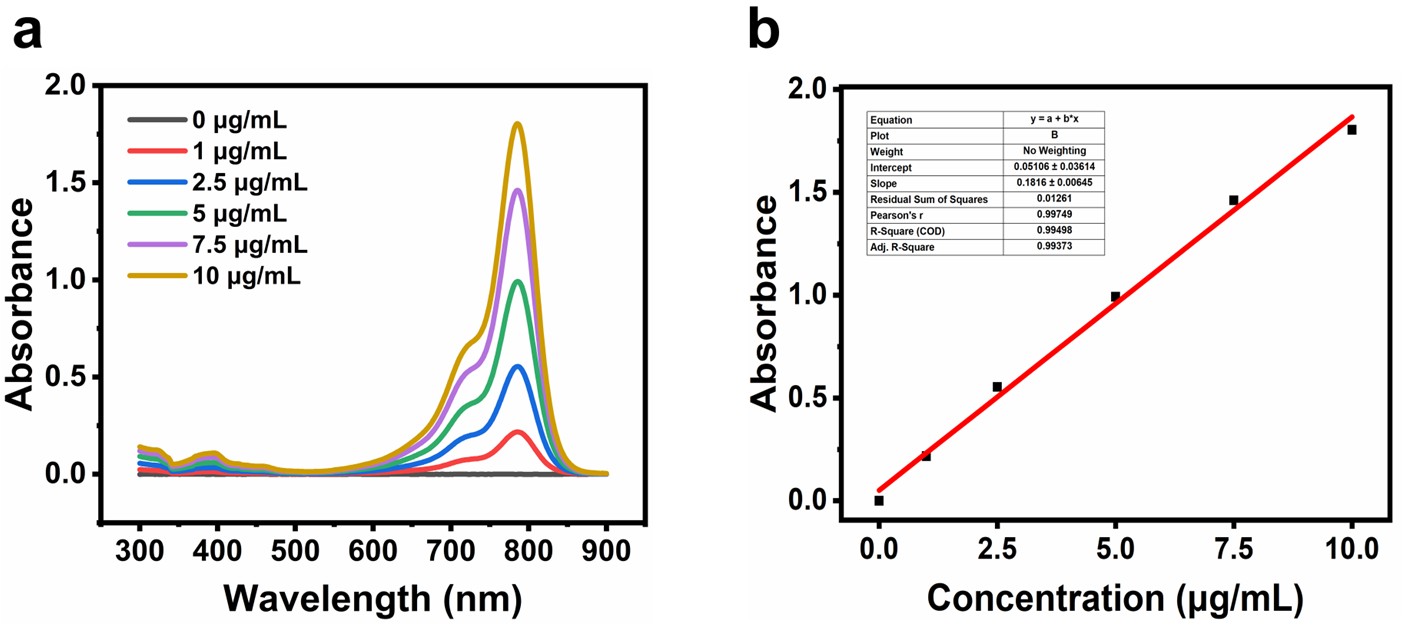


Figure S5. (a) UV/Vis absorption spectra of ICG-SH with different concentration.

(b) The standard curve of ICG-SH according to the absorption.


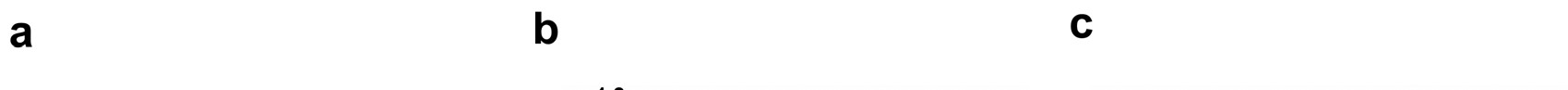

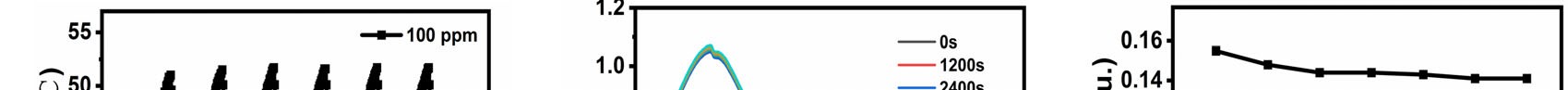

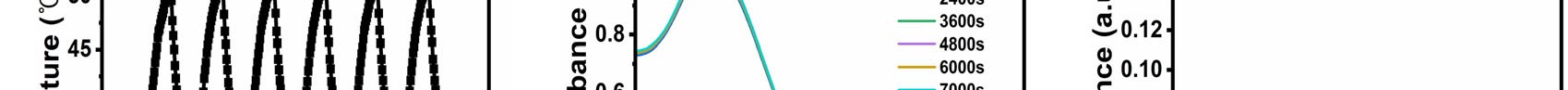

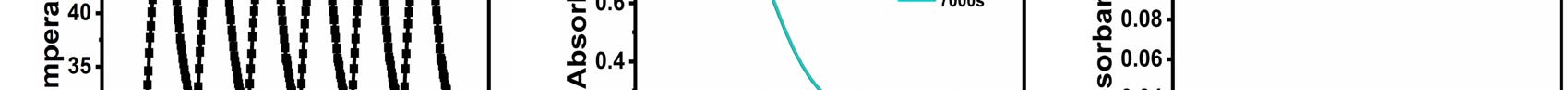

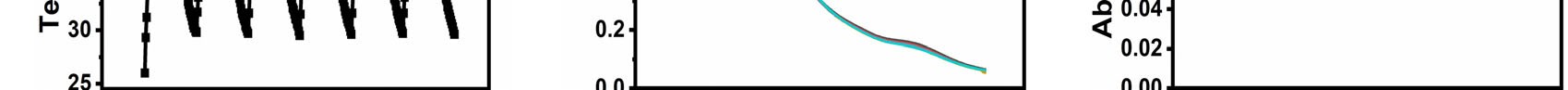

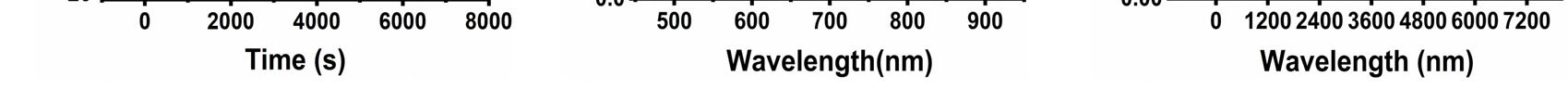


Figure S6. (a) Photothermal curves of AIG nanoparticles subjected to six 808 nm laser irradiation on/off cycles at 1.0 W/cm^2^. (b) UV-Vis absorption spectrum of an AIG solution over six cycles of heating and natural cooling. (c) The plot of the UV-Vis absorption value of AIG at 808 nm changes with the irradiation time.


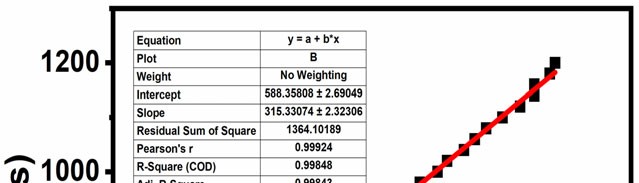

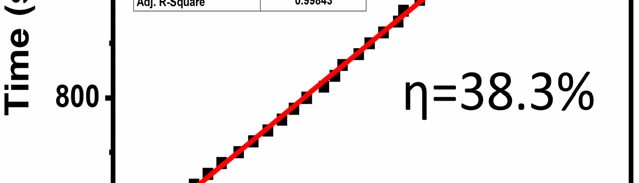

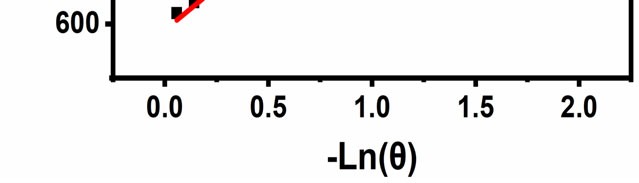


Figure S7. Plot of cooling time versus negative natural logarithm of the temperature driving force.


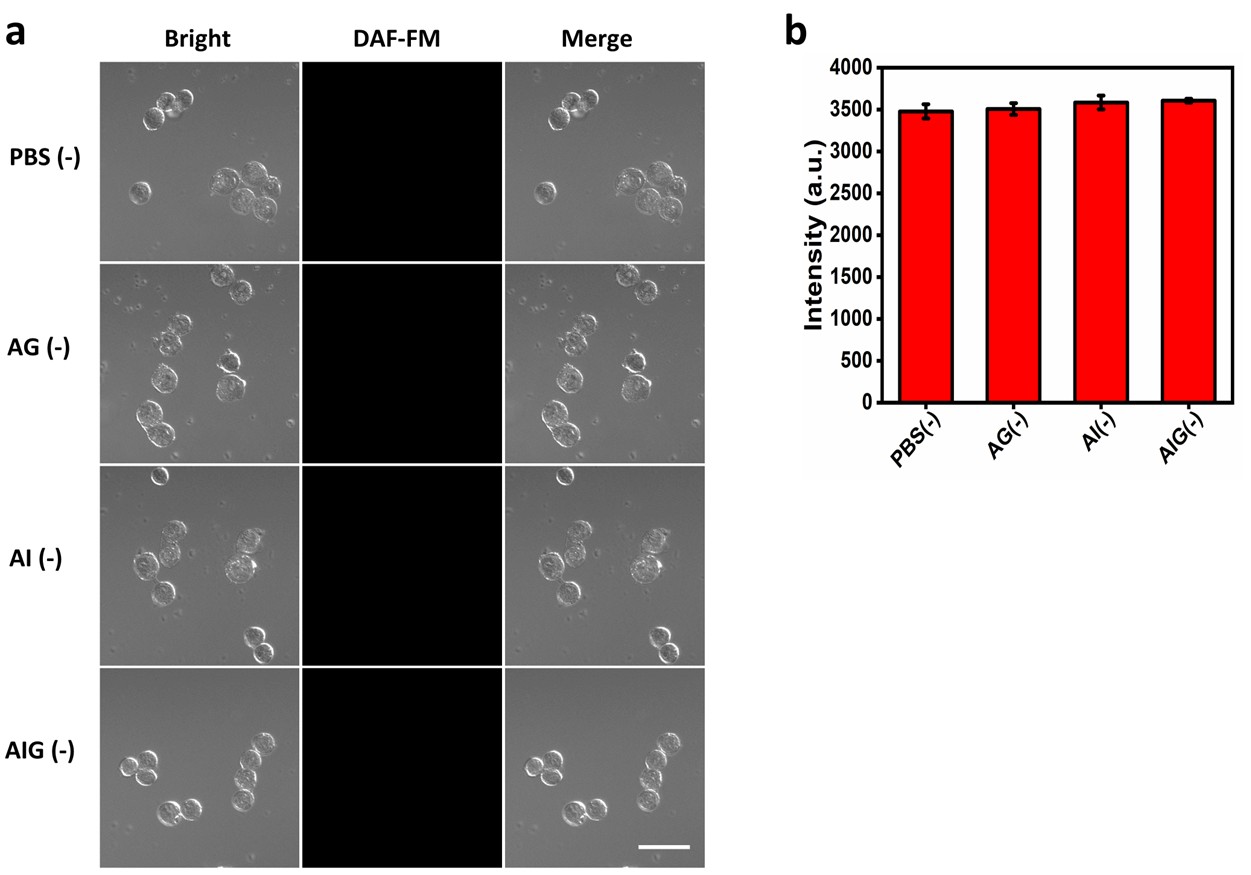


Figure S8. (a) Intracellular NO detection of MC38 cells after co-incubation with AG, AI and AIG under dark conditions. Scale bar: 40 μm. (b) Average cellular fluorescence signal of DAF-FM DA indicating NO under dark conditions in each group.


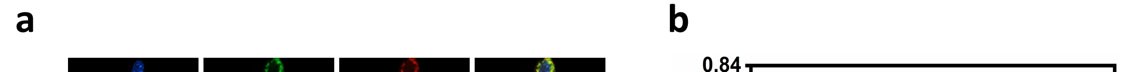

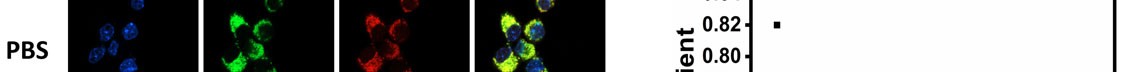

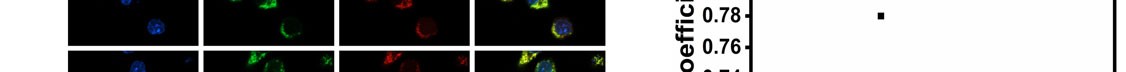

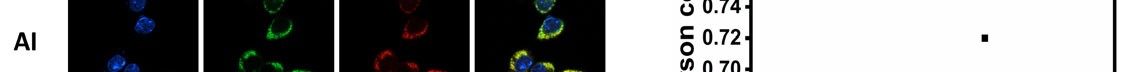

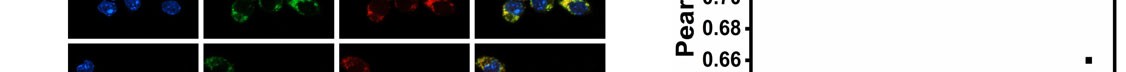

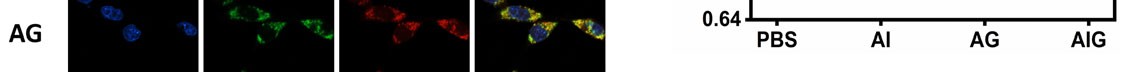

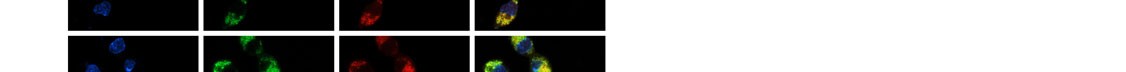

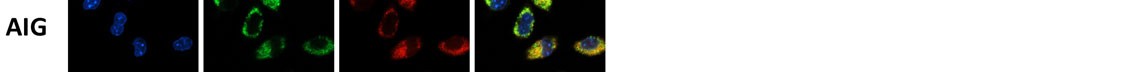

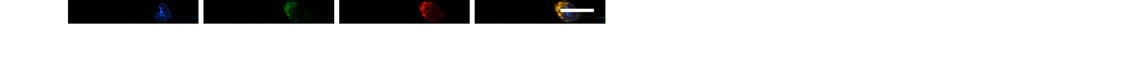

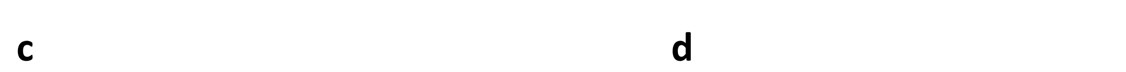

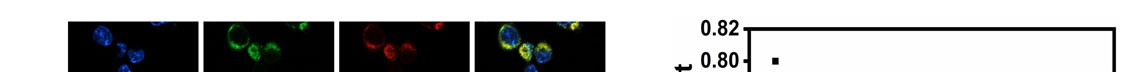

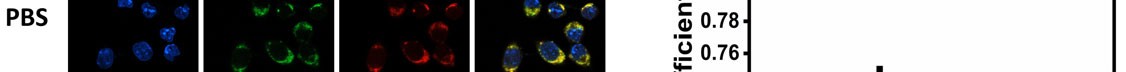

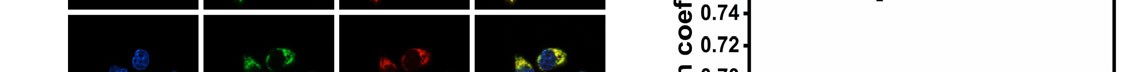

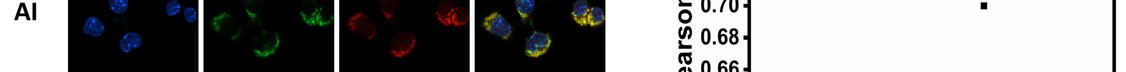

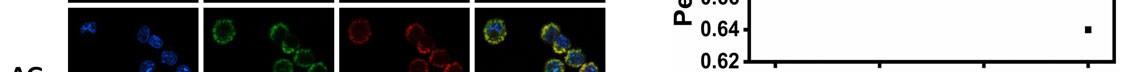

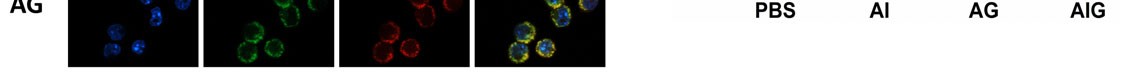

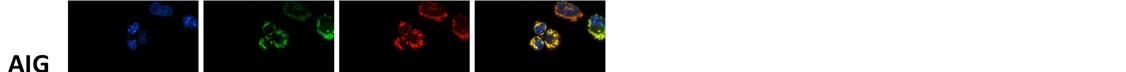

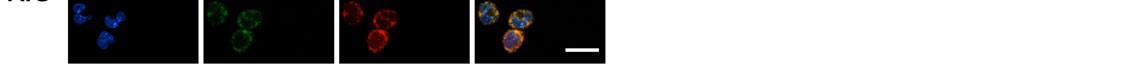


Figure S9. The images of repeated cytochrome c release (a, c) and Pearson coefficient data (b, d). Scale bar: 40 μm.


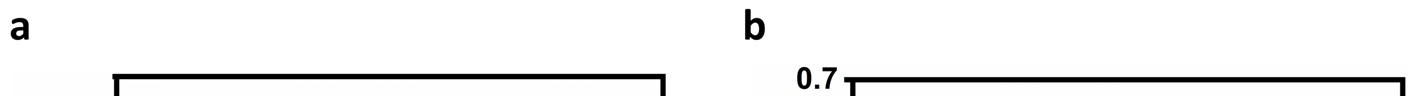

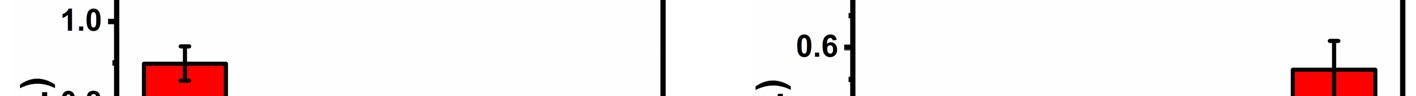

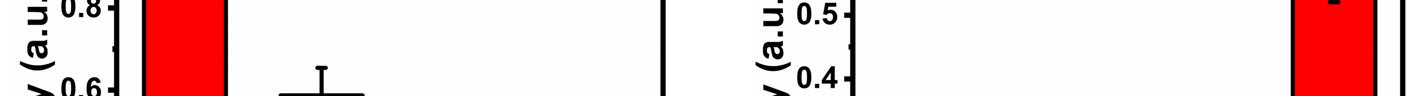

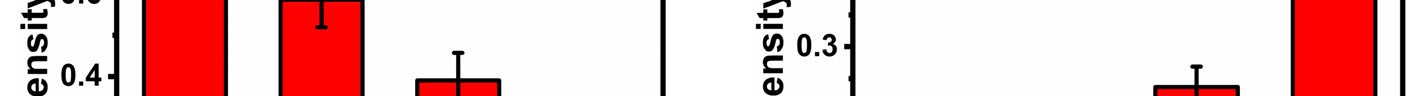

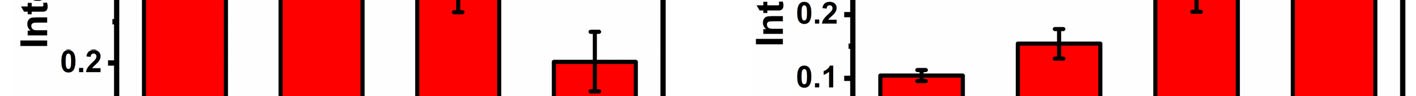

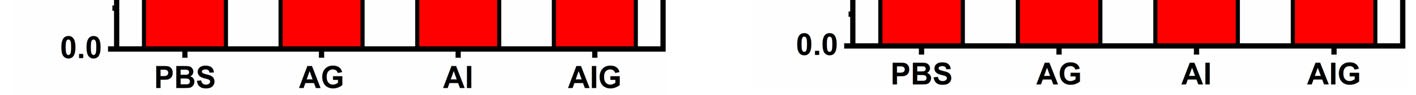


Figure S10. The average cellular bcl-2 (a) and cleaved caspase-3 (b) expression signal of each group.


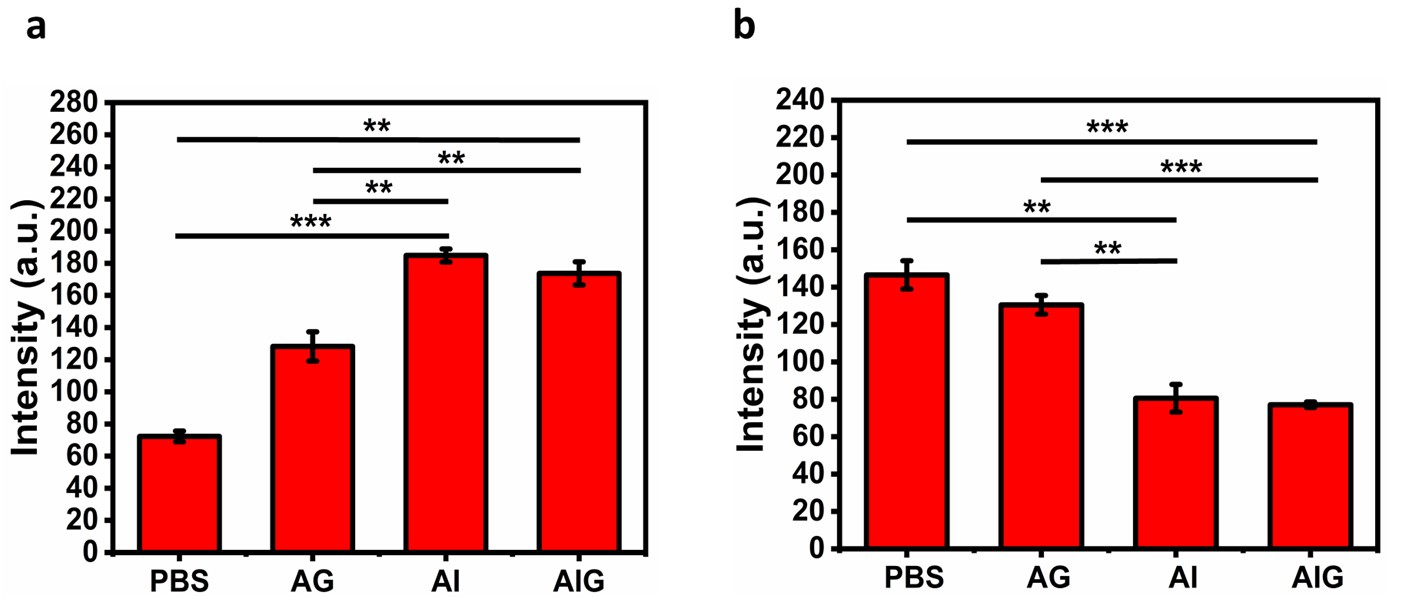


Figure S11. (a) Quantitative analysis of the level of CRT. (b) Quantitative analysis of the level of intranuclear HMGB1. *p < 0.05, **p < 0.01, ***p < 0.001.


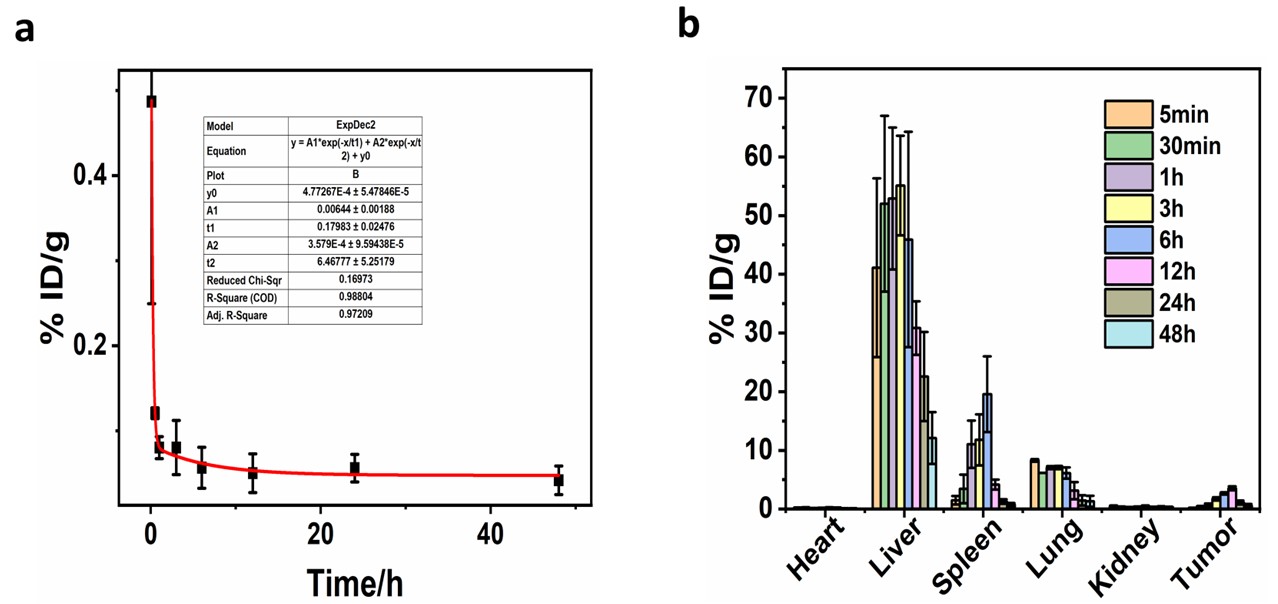


Figure S12. (a) *In vivo* pharmacokinetics of AIG. (b) Biodistributions of AIG different time after injection.


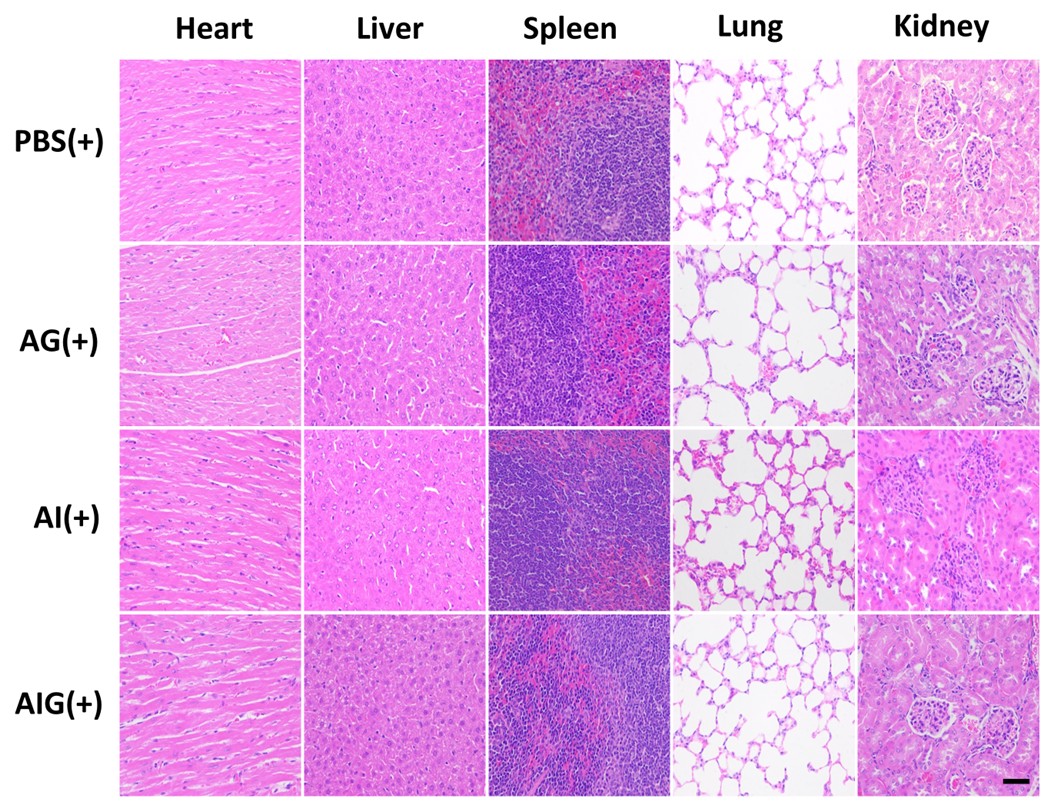


Figure S13. H&E stained tissues of heart, liver, spleen, lung and kidney of mice from the PBS, AG, AI and AIG under 808 nm laser irradiation. Scale bar: 50 μm.


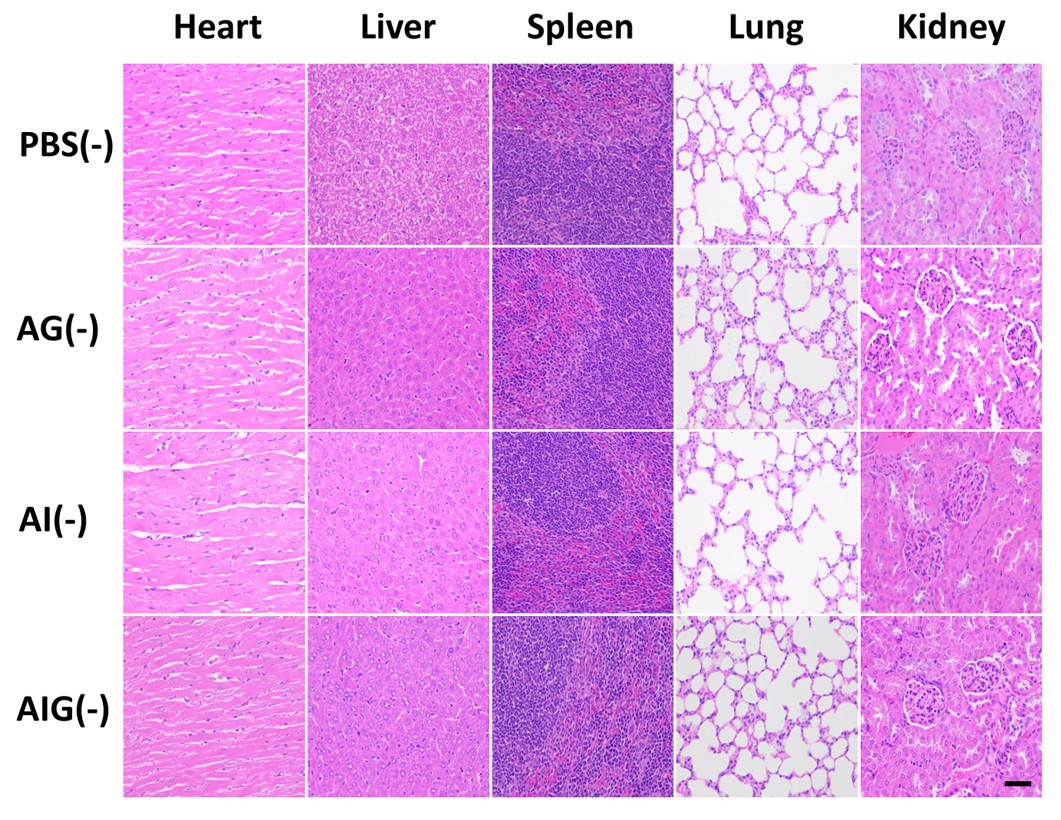


Figure S14. H&E stained tissues of heart, liver, spleen, lung, and kidney of mice from the PBS, AG, AI and AIG without NIR laser irradition. Scale bar: 50 μm.


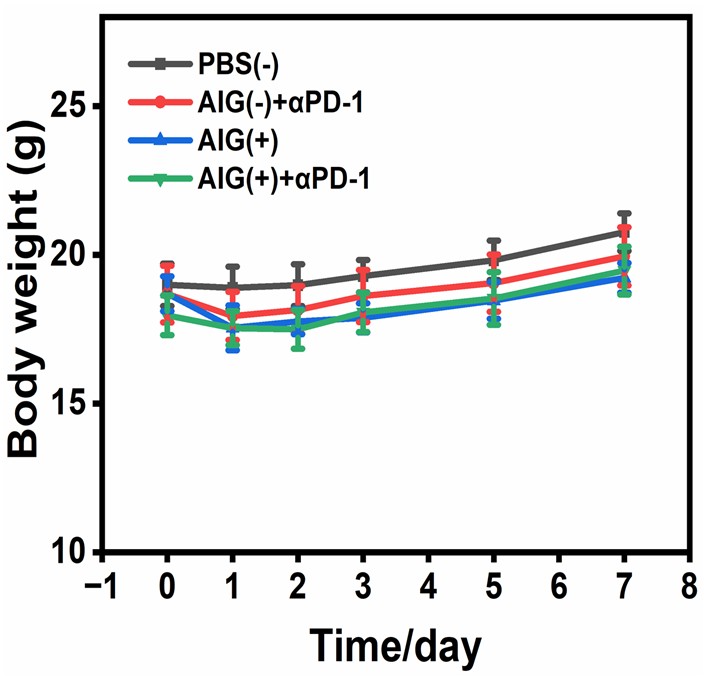


Figure S15. Body weight curves of mice.


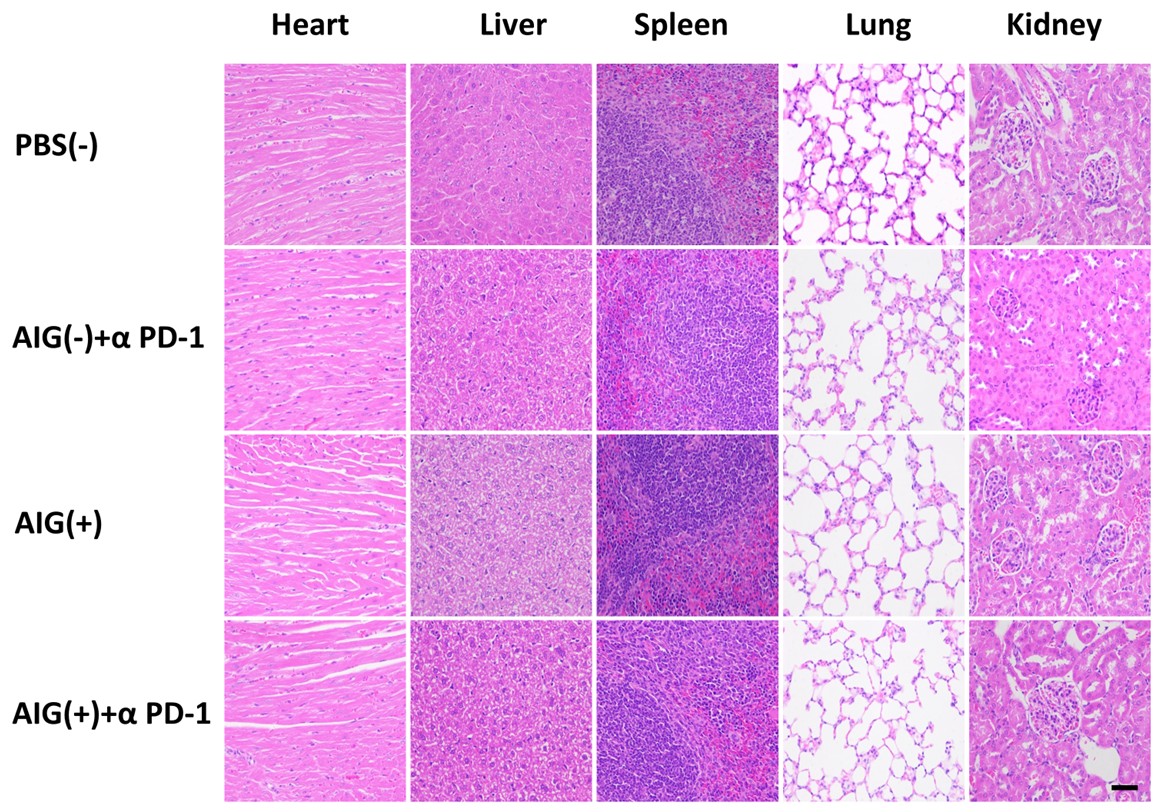


Figure S16. H&E stained tissues of heart, liver, spleen, lung, and kidney of mice from the PBS(-), AIG(-)+α PD-1, AIG(+) and AIG(+)+α PD-1. Scale bar: 50 μm.

Table S1. The quantitative analysis of loading efficiency of ICG and GSNO and molar ratio of Au, ICG and GSNO.

|  | Loading efficiency of ICG  (%) | Loading efficiency of GSNO (%) | n(Au):n(ICG):n(GSNO) |
| --- | --- | --- | --- |
| Au-GSNO(AG) | / | 12.94 ± 4.51 | 26:0:1 |
| Au-ICG(AI) | 32.52 ± 1.76 | / | 9:1:0 |
| Au-ICG-GSNO(AIG) | 33.83 ± 2.14 | 10.33 ± 4.51 | 160:18:5 |
